# Levocarnitine improves cardiac energy metabolic remodeling in myocarditis mice

**DOI:** 10.3389/fphar.2025.1706936

**Published:** 2026-01-09

**Authors:** Shutong Yang, Xiaoou Li, Zhenpeng Lu, Xiang Xu, Zuzhen Guo, Bing He

**Affiliations:** 1 Department of Pediatrics, Renmin Hospital of Wuhan University, Wuhan, Hubei, China; 2 Zuoling Street Community Health Service Center, Wuhan, Hubei, China

**Keywords:** experimental autoimmune myocarditis, levocarnitine, metabolic remodeling, mitochondrion, PI3K/AKT

## Abstract

**Introduction:**

Energy metabolic remodeling represents a critical pathological mechanism in myocarditis progression. Levocarnitine (LC), an essential cofactor for fatty acid oxidation, demonstrates potential in modulating cardiac metabolism. This study investigated the therapeutic effects of LC on myocardial energy metabolic remodeling and explored the underlying molecular mechanisms.

**Methods:**

The experimental autoimmune myocarditis (EAM) mouse model was constructed using α-myosin. Cardiac function, myocardial inflammatory infiltration, and mitochondrial structure were evaluated using echocardiography, HE staining, and transmission electron microscopy, respectively. Metabolic parameters including free fatty acid (FFA), lactic acid (LAC), mitochondrial complex IV (COX IV) activity, and adenosine triphosphate (ATP) levels were measured using colorimetry. Serum heart-type fatty acid-binding protein (H-FABP) levels were measured by ELISA, and reactive oxygen species (ROS) levels were determined by flow cytometry. The expression of organic carnitine transporter type 2 (OCTN-2) and carnitine palmitoyltransferase-1B (CPT-1B) were determined by Western blot. Furthermore, network pharmacology and molecular docking were employed to predict the therapeutic targets and mechanisms of LC in myocarditis. The activity of the phosphatidylinositol-3-kinase/protein kinase B (PI3K/Akt) pathway and the expression of peroxisome proliferator-activated receptor γ coactivator-1α (PGC-1α) were verified by Western blot.

**Results:**

LC treatment significantly improved cardiac function and attenuated myocardial inflammatory infiltration in EAM mice. It ameliorated mitochondrial structural damage, enhanced COX IV activity and ATP production, and reduced the accumulation of FFA, LAC and ROS in myocardial tissues. It also lowered serum H-FABP levels while upregulating the expression of OCTN-2 and CPT-1B. Combining network pharmacology and molecular docking, Akt was identified as the key therapeutic target of LC in cardiomyopathy and demonstrated good binding affinity with LC. *In vivo* validation confirmed that LC decreased Akt phosphorylation in the myocardium of EAM mice, while PGC-1α expression increased.

**Conclusion:**

LC effectively improved myocardial metabolic remodeling and alleviated cardiac insufficiency in myocarditis. The underlying mechanism may involve LC-mediated suppression of the PI3K/Akt signaling pathway, potentially linked to increased expression of the key mitochondrial regulator PGC-1α.

## 1 Introduction

Myocarditis is an inflammatory cardiomyopathy characterized by focal or diffuse inflammatory cell infiltration within the myocardium, displaying heterogeneous clinical features of severity. While mild cases usually show self-limitation, susceptible individuals may develop pathological myocardial hypertrophy, fibrosis, and eventually progress to dilated cardiomyopathy (DCM) (Sur et al., 2024; D’Elia et al., 2024). Therefore, conducting a comprehensive study of myocarditis pathogenesis and developing effective therapeutic strategies are essential for enhancing cardiac function and improving long-term clinical outcomes.

Recent studies have shown that, as the cell with the highest density of mitochondria, impaired energy metabolism in cardiomyocytes is a key pathological basis of damaged myocardium, which brings about a series of significant changes in myocardial energy and substrate metabolism, leading to “metabolic remodeling,” where the myocardium preferentially shifts substrates from fatty acids to glucose for utilization, and involves abnormalities in oxidative phosphorylation and a decrease in high-energy phosphates, resulting in dysfunctions in myocardial function and structure (van Bilsen et al., 2004). Existing evidence suggests that myocardial metabolic remodeling plays a crucial role in the pathological process of myocarditis, representing a key stage in the progression of myocardial injury from compensation to decompensation, and is closely associated with the development of DCM due to myocarditis (Remels et al., 2018; Mohamud et al., 2023). Therefore, optimizing myocardial substrate utilization and modulating mitochondrial function through metabolic drugs, thereby ameliorating the adverse progression of metabolic remodeling, is a key approach in the clinical management of myocarditis.

LC is an essential compound that facilitates fatty acid metabolism, supports mitochondrial import of fatty acids through OCTN2 on the outer mitochondrial membrane, and regulates the balance between fatty acid and glucose oxidative energy supply, thereby promoting mitochondrial energy metabolism (Longo et al., 2016). Additionally, LC functions as an antioxidant and anti-inflammatory agent that scavenges oxygen-free radicals, influences mitochondrial autophagy, and preserves mitochondrial membrane integrity (Wang et al., 2018; Elantary and Othman, 2024). Several studies have confirmed the cardioprotective effects of LC, and most current mechanistic research has concentrated on cardiovascular diseases such as diabetic cardiomyopathy, coronary artery disease, and arrhythmia (Li et al., 2023; Farag et al., 2024; Zhang et al., 2021). However, the regulatory mechanism of LC in the metabolic remodeling of myocarditis has not yet been clarified. In this study, we investigated the effect of LC on energy metabolic remodeling in myocarditis and explored the potential signaling pathways by establishing an animal model of EAM, combined with network pharmacology and molecular docking.

## 2 Materials and methods

### 2.1 Animal experiment

#### 2.1.1 Animals

Twelve male Balb/c mice, aged 6–8 weeks and weighing 20–25 g, with SPF grade, were purchased from the Animal Experiment Centre of Three Gorges University, with production license No. SCXK (E) 2022-0012. The mice used in this experiment were approved by the Ethics Committee of Animal Experimentation of the People’s Hospital of Wuhan University (No. 20240308).

Twelve Balb/c mice were randomly divided using a random number table into the control group (Control group, n = 4), the experimental autoimmune myocarditis group (EAM group, n = 4), and the levocarnitine-treated group (LC group, n = 4), and housed at room temperature in the SPF animal house. MyHC-α peptide was dissolved in 0.9% sterile NaCl solution, and a turbid emulsion was obtained by mixing α-myosin heavy chain peptide (P31501, Sangon, Shanghai, China) with Complete freund’s adjuvant (HY-P73565, MCE, USA) in a 1:1 volume ratio using a tee-tube. Mice in the EAM and LC groups received subcutaneous injections into the dorsal and axillary lymph nodes on days 0 and 7, respectively, with 200 µL of emulsion containing 200 µg of peptide per mouse. The Control group was injected with an equal amount of saline subcutaneously at the same sites. LC (01723120008, Northeast Pharmaceutical Group Co., Ltd., China) was administered by gavage at 200 mg/kg on day 8 for 2 weeks in the LC group, following modeling, while the Control and EAM groups received an equal volume of saline on day 8. On day 21, mice were anesthetized by intraperitoneal injection of 2.5% tribromoethanol followed by retro-orbital blood collection, euthanasia via cervical dislocation, and heart tissue harvest and weighing.

#### 2.1.2 Echocardiography

Cardiac echocardiography was performed on day 21 in mice anesthetized with 2% isoflurane gas and probed by an ultra-high resolution small animal ultrasound imaging system (SigmaVET, Esaote, Italy). The left ventricular ejection fraction (LVEF), fractional shortening (FS), left ventricular end-diastolic internal dimension (LVIDd), left ventricular end-systolic internal dimension (LVIDs), and left ventricular posterior wall thickness (LVPW) were measured to assess left ventricular function.

#### 2.1.3 Histopathology

Mouse heart tissue was collected and fixed in 4% paraformaldehyde solution, then processed through gradient ethanol dehydration, embedded in paraffin, sectioned, dewaxed, stained with hematoxylin-eosin, and sealed with neutral gum. Myocardial histopathological changes were observed under a light microscope. Three fields of view were randomly selected under high magnification (×200). The inflammation area was calculated as the ratio of the inflamed area to the total section using ImageJ software. The scoring criteria were as follows (Lema et al., 2024): score 0: no inflammatory cell infiltration; score 1: inflammatory infiltration area <10%; score 2: inflammatory infiltration between 10% and 30%; score 3: inflammatory infiltration area between 30% and 50%; score 4: inflammatory infiltration >50%.

#### 2.1.4 Electron microscopy observation of mitochondrial structure

Mouse heart tissues were fixed with 2.5% glutaraldehyde for 24 h, then treated with 1% osmium sodium phosphate buffer for 2 h. The tissues were dehydrated through an ethanol gradient, embedded in epoxy resin, and cut into ultrathin sections. The mitochondrial ultrastructure was observed under a transmission electron microscope (HT7700-SS, Hitachi, Japan) after double staining with saturated aqueous uranyl acetate and lead citrate.

#### 2.1.5 Detection of substrate metabolism

Blood was taken from the mouse’s eye, centrifuged at 4 °C at 3,000 rpm for 10 min, and the supernatant was collected to measure H-FABP activity. Follow the instructions in the H-FABP ELISA kit (JM-04323H2, Jingmei, Jiangsu, China) manual to determine H-FABP levels in each group’s serum using a multifunctional enzyme analyzer (Molecular Devices, Shanghai, China). Myocardial tissues were collected, ground with PBS in an ice bath, and then centrifuged at 3,000 rpm for 10 min. The supernatant was used to measure FFA (A042-1-1, Jiancheng, Nanjing, China) and LAC (A019-2-1, Jiancheng, Nanjing, China) in the cardiac tissues of each group, following the kit’s instructions.

#### 2.1.6 Mitochondrial function assay

The mitochondrial suspension was obtained from heart tissue using the mitochondrial extraction kit (G006-1-1, Jiancheng, Nanjing, China). It was then placed in a boiling water bath for 10 min. Afterwards, the homogenate was centrifuged at 3,000 rpm for 10 min. The supernatant was collected to test the ATP content, following the procedures of the ATP content test kit (A095-1-1, Jiancheng, Nanjing, China). Mitochondrial complex IV activity was measured with the kit (E-BC-K837-M, Elabscience, Wuhan, China) to assess the integrity of the mitochondrial respiratory chain.

#### 2.1.7 Reactive oxygen species

ROS content was detected using a kit (S0033S, Beyotime, Shanghai, China). Cardiac tissues were cut and broken down with the tryptic digest, and the cell sieve was filtered into a single-cell suspension. Cardiomyocytes were centrifuged at 1,200 rpm for 5 min to collect the precipitate. A 10 μmol/L DCFH-DA probe was added and incubated in the dark at 37 °C for 20 min. The cells were washed, resuspended, and analyzed by flow cytometry (Afla Aesar, Beckman, USA). The fluorescence intensity was analyzed using FlowJo software to evaluate the ROS level.

#### 2.1.8 Western blot

Total protein was extracted by adding heart tissue blocks to the PIPA protein lysate prepared according to the ratio, and protein concentration was measured using the BCA method. 40 μg of total protein from each sample were loaded for SDS-PAGE electrophoresis and transferred to a PVDF membrane. The PVDF membrane was soaked in 5% skim milk and incubated at room temperature for 2 h. Primary antibodies—GAPDH (60004-1-Ig, Proteintech, Wuhan, China), CPT1B (DF3904, Affinity, USA), OCTN2 (16331-1-AP, Proteintech, Wuhan, China), Akt (60203-2-Ig, Proteintech, Wuhan, China), p-Akt (80642-1-rr, Proteintech, Wuhan, China), and PGC-1α (Ab 313559, Abcam, Shanghai, China)—were incubated at 4 °C overnight. The membrane was washed with TBST, then incubated at room temperature for 2 h with horseradish peroxidase-labeled goat anti-rabbit secondary antibody (A0208, Beyotime, Shanghai, China) and goat anti-mouse secondary antibody (SA00001-1, Proteintech, Wuhan, China). After washing again with TBST, ECL was added to develop the signal. ImageJ software was used for analysis, and the ratio of the gray value of the target protein bands (CPT1B, OCTN2, Akt, p-Akt, and PGC-1α) to the internal reference protein (GAPDH) was calculated as the relative expression of the target protein.

### 2.2 Network pharmacology and molecular docking

#### 2.2.1 Drug targets of levocarnitine

The SMILES and structural formulas of LC were obtained from the PubChem database (https://pubchem.ncbi.nlm.nih.gov/), and then SwissTargetPrediction (http://swisstargetprediction.ch/), PharmMapper (https://lilab-ecust.cn/pharmmappe r/index.html), and CTD (https://ctdbase.org/) databases were used to identify LC-related targets. The data from these databases were integrated, and duplicates were removed.

#### 2.2.2 Targets of myocarditis

GeneCards (https://www.genecards.org), OMIM (https://mirror.omim.org), and Disgenet (https://disgenet.com/) databases were used to search for disease-related targets using the keyword “myocarditis,” and duplicate targets were then removed after identifying the disease-related targets.

#### 2.2.3 Obtaining intersecting targets for LC treatment of myocarditis

The LC drug action targets and myocarditis-related targets were imported into the Venny2.1.0 online platform (https://bioinfogp.cnb.csic.es/tools/venny/) together, and their intersection was identified to find the common targets. A Venn diagram was then plotted to visualize the potential targets of LC for myocarditis.

#### 2.2.4 Construction of protein-protein interaction network

Imported the intersection target into the STRING 12.0 database (https://cn.string-db.org/), selected “Multiple proteins” and the species as “*Homo Sapiens*”, the confidence threshold was set at 0.4. Mapped the protein-protein interaction network (PPI). The results were imported into CytoScape 3.10.0 and analyzed using the plug-in CytoNCA for Closeness centrality (CC), Betweenness centrality (BC), and Degree centrality (DC). The key target genes were identified with median values exceeding each topology measure, and the top 10 proteins were finally selected as core targets through repeated screening.

#### 2.2.5 Gene ontology (GO) enrichment analysis and kyoto encyclopedia of genes and genomes (KEGG) pathway enrichment analysis

The common targets of levocarnitine and myocarditis were uploaded to the DAVID database (https://davidbioinformatics.nih.gov/), and GO and KEGG analyses were performed to obtain the biological process (BP), cellular component (CC), molecular function (MF), and related signaling pathways, with P < 0.01 as the cut-off point. Visual analysis was conducted through the microbiological letter platform (https://www.bioinformatics.com.cn/).

#### 2.2.6 Molecular docking

**FIGURE 1:**
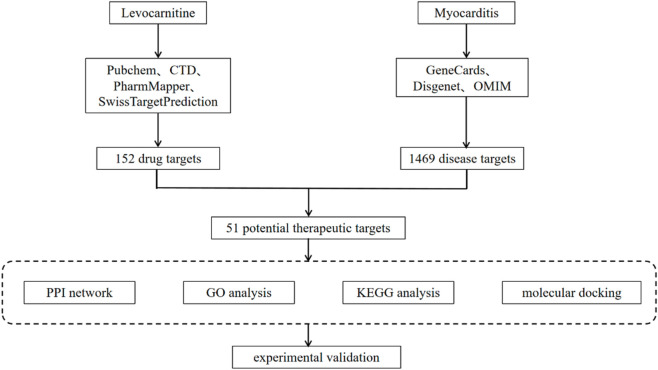
Workflow of the study based on network pharmacology and molecular docking aimed at exploring the mechanism of levocarnitine in treating myocarditis.

Proteins encoded by the core target genes were selected as molecular docking proteins, with LC serving as the binding ligand. Protein structures were obtained from the PDB database (https://www.rcsb.org/) as PDB format files. The 3D structure of the ligand LC was downloaded from the PubChem database and saved in SDF format. Molecular docking was carried out using the CB-Dock2 online platform (https://cadd.labshare.cn/cb-dock2/php/blinddock.php). CB-Dock2 operates in blind docking mode, employing a curvature-based cavity detection algorithm to automatically identify potential binding regions on the protein surface and determine the center coordinates and dimensions of the binding pockets (Liu et al., 2022). Subsequently, AutoDock Vina is invoked for precise molecular docking and conformational sampling. The platform automatically preprocesses the protein structures (hydrogenation, side-chain completion, removal of water and heteroatoms) and ligand (charge addition, 3D conformation generation). The minimum Vina score was used as the criterion to evaluate binding activity, with more negative values indicating greater docking stability. The molecular docking results were visualized using Pymol software. The flowchart of network pharmacology and molecular docking in this study is shown in Figure 1.

### 2.3 Statistical analysis

GraphPad Prism 10 was used for data analysis. The Shapiro-Wilk test was used to verify data normality. Multiple-group comparisons were analyzed by one-way ANOVA, followed by Tukey’s *post hoc* test. The Pearson correlation coefficient was used to analyze the correlation between two variables. The experimental data were expressed as mean ± standard error of the mean (Mean ± SEM). P < 0.05 was considered statistically significant.

## 3 Results

### 3.1 Animal experiment

#### 3.1.1 LC improved cardiac function and reduced myocardial inflammation in EAM mice

**FIGURE 2:**
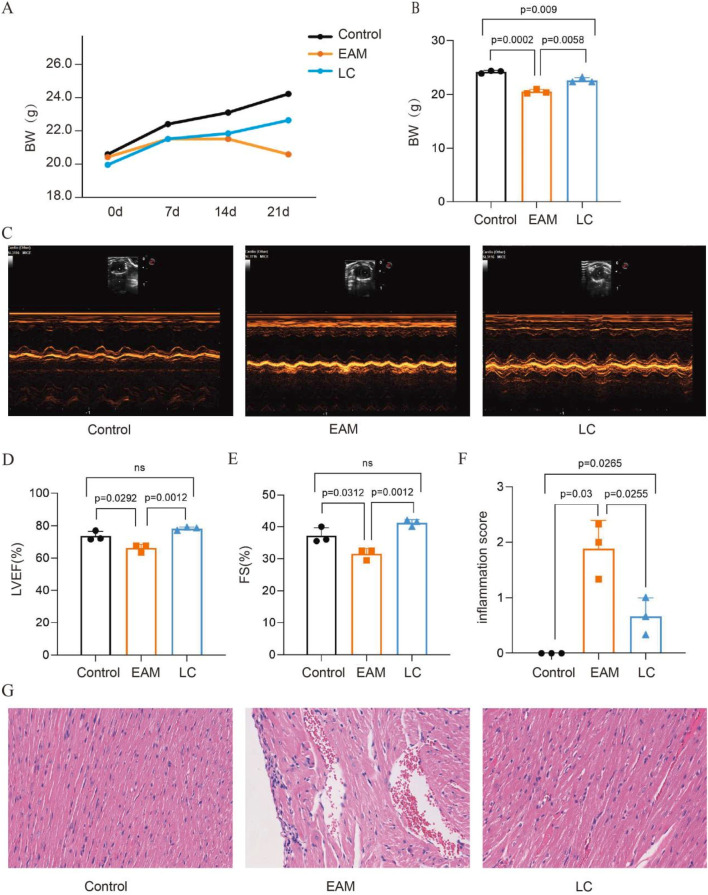
LC improves cardiac insufficiency and reduces myocardial inflammatory infiltration in EAM mice. **(A)** Changes in body weight and **(B)** body weight of mice in each group on day 21, n = 3. **(C)** Parasternal left ventricular short-axis M-mode echocardiograms of mice in each group on day 21. **(D)** Changes in left ventricular ejection fraction and **(E)** left ventricular shortening fraction, n = 3. **(F)** Myocardial inflammatory infiltration scores in each group of mice, n = 3. **(G)** HE staining showing inflammatory lesions in cardiac tissue (×200). Data are expressed as Mean ± SEM with individual data points shown. LVEF, left ventricular ejection fraction; FS, fractional shortening.

Mice in the EAM group showed varying degrees of hair dishevelment and dullness, along with reduced food intake. Some mice experienced loose stools and gradually lost weight. In contrast, mice in the LC group also lacked luster but demonstrated improved food intake and gained weight compared to the EAM mice (Figure 2A). By day 21, the LC group significantly alleviated weight loss in the EAM mice (P < 0.05) (Figure 2B). Subsequently, cardiac ultrasonography revealed no significant differences between groups in LVIDd, LVIDs, and LVPW (Table 1). However, LVEF and FS were significantly lower in the EAM group, indicating that LC alleviated cardiac insufficiency in the EAM mice (P < 0.05) (Figures 2C–E). HE staining showed disorganization of cardiomyocytes in the EAM group, with numerous inflammatory cell infiltrates, while LC intervention markedly reduced inflammation in cardiac tissue (Figure 2G). Inflammation scores also showed higher pathological scores in the EAM group compared to the Control group, with a reduction observed in the LC group (P < 0.05) (Figure 2F). In conclusion, LC exerts a cardioprotective effect in EAM mice.

**TABLE 1:** Cardiac function parameters obtained from echocardiography.

Groups	LVEF (%)	FS (%)	LVIDd (mm)	LVIDs (mm)	LVPW (mm)
Control	73.5 ± 3.04	37.17 ± 2.47	3.67 ± 0.08	2.3 ± 0.13	1.22 ± 0.08
EAM	66.17 ± 2.31*	31.5 ± 1.73*	3.73 ± 0.19	2.55 ± 0.22	1.18 ± 0.23
LC	74.17 ± 7.94^##^	41.17 ± 1.04^##^	3.72 ± 0.2	2.3 ± 0.2	1.16 ± 0.11

Data are presented as mean ± SEM; *p < 0.05. **p < 0.01 vs. Control; #p < 0.05. ##p < 0.01 vs. EAM. LVEF, left ventricular ejection fraction; FS, fractional shortening; LVIDd, left ventricular end-diastolic internal dimension; LVIDs, left ventricular end-systolic internal dimension; LVPW, left ventricular posterior wall thickness.

#### 3.1.2 LC attenuated myocardial mitochondrial structural damage and respiratory chain abnormalities in EAM mice

**FIGURE 3:**
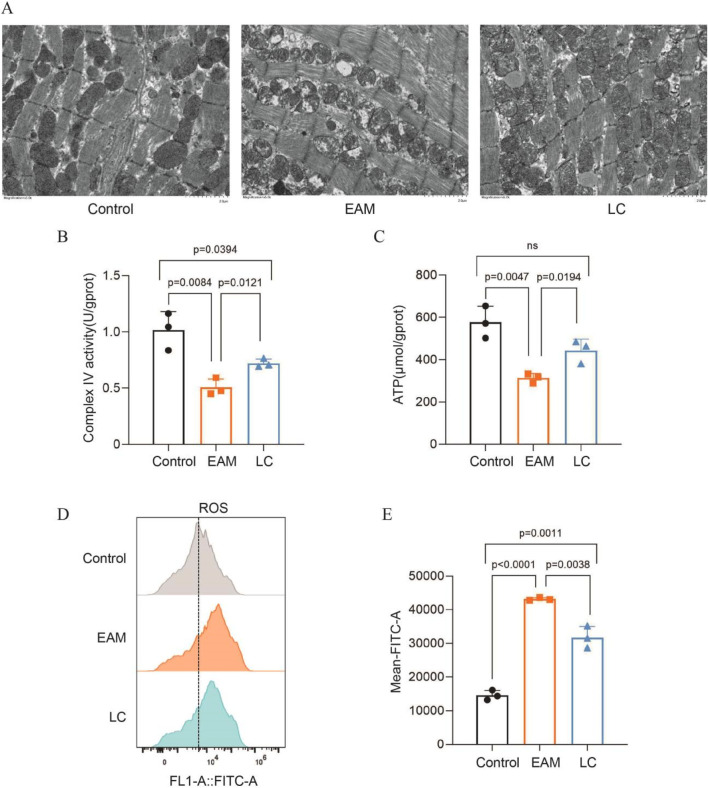
LC attenuated myocardial mitochondrial structural damage and respiratory chain abnormalities in EAM mice. **(A)** Transmission electron microscopy showing morphological changes in mouse heart mitochondria (×5,000, scale bar = 2 μm). Changes in **(B)** mitochondrial complex IV activity and **(C)** ATP levels in each group, n = 3. **(D,E)** DCFH-DA staining flow cytometry to detect ROS levels in each group, n = 3. Data are expressed as Mean ± SEM with individual data points shown. ATP, adenosine triphosphate; ROS, reactive oxygen species.

Transmission electron microscopy observed the mitochondrial ultrastructure. In the control group, myocardial fibers were neatly organized, with clear Z lines, elliptical mitochondria, intact inner and outer membranes, and well-defined dense mitochondrial cristae. Conversely, the EAM mice displayed fragmented and sparse myofibrillar architecture, with rounded mitochondria, loss of inner membrane integrity, fragmented cristae, and swollen matrix. LC attenuated the structural damage of the myocardial mitochondria in the EAM mice (Figure 3A). To further determine whether LC alleviated cardiac mitochondrial respiratory dysfunction in the EAM mice, we evaluated mitochondrial performance through three parameters: respiratory chain enzyme activity, energy production, and oxidative stress. COX IV activity and ATP levels were significantly decreased in the EAM mice. After LC treatment, both COX IV activity (Figure 3B) and ATP levels (Figure 3C) increased significantly. Flow cytometry results revealed that ROS levels were elevated in the EAM group and significantly decreased after LC intervention (P < 0.05) (Figures 3D,E). These findings suggest that mitochondrial structure and the oxidative respiratory chain were impaired in myocarditis mice, with decreased ATP production and increased oxidative stress, while LC appears to protect mitochondrial structure, boost respiratory chain enzyme activity, improve energy metabolism, and lower oxidative stress.

#### 3.1.3 LC improved the utilization of myocardial mitochondrial substrates in EAM mice

**FIGURE 4:**
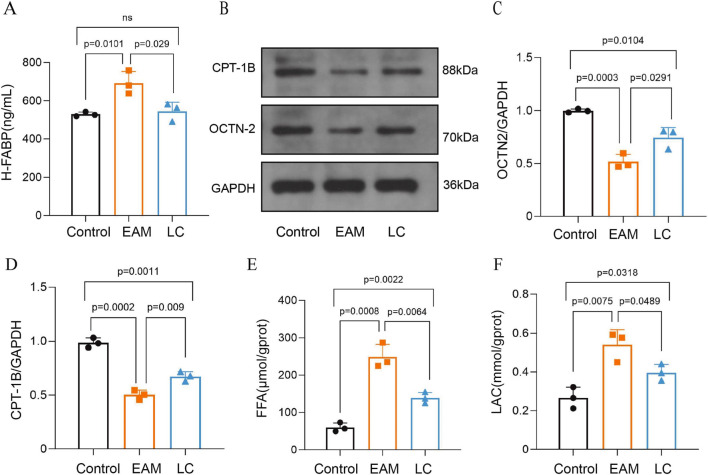
LC improves myocardial mitochondrial substrate utilization in EAM mice. **(A)** Serum H-FABP levels in each group of mice, n = 3. **(B–D)** Western blot analysis of OCTN-2 and CPT-1B proteins, n = 3. **(E)** FFA and **(F)** LAC levels in myocardial tissue from each group of mice, n = 3. Data are expressed as Mean ± SEM with individual data points shown. FABP, heart-type fatty acid-binding protein; OCTN2, organic carnitine transporter novel type 2; CPT-1B, carnitine palmitoyltransferase-1B; FFA, free fatty acid; LAC, lactic acid.

Compared to the Control group, serum H-FABP levels were higher in the EAM group, and myocardial OCTN-2 and CPT-1B expression were significantly lower. LC intervention significantly attenuated serum H-FABP release while upregulating cardiac OCTN-2 and CPT-1B protein levels (P < 0.05) (Figures 4A–D). Additionally, myocardium FFA and LAC levels in the EAM group were significantly elevated. After LC administration, FFA and LAC accumulation decreased, with statistically significant differences compared to the EAM group (P < 0.05) (Figures 4E,F). These results suggest that EAM-induced myocardial injury resulted in the release of H-FABP into the circulation, disrupted fatty acid transport, decreased β-oxidation, and enhanced anaerobic glycolysis. Meanwhile, LC promoted fatty acid utilization, decreased glucose metabolism, and improved cardiomyopathic energy substrate utilization in myocarditis mice.

### 3.2 Network pharmacology and molecular docking

#### 3.2.1 Acquisition and enrichment analysis of common targets between LC and myocarditis

**FIGURE 5:**
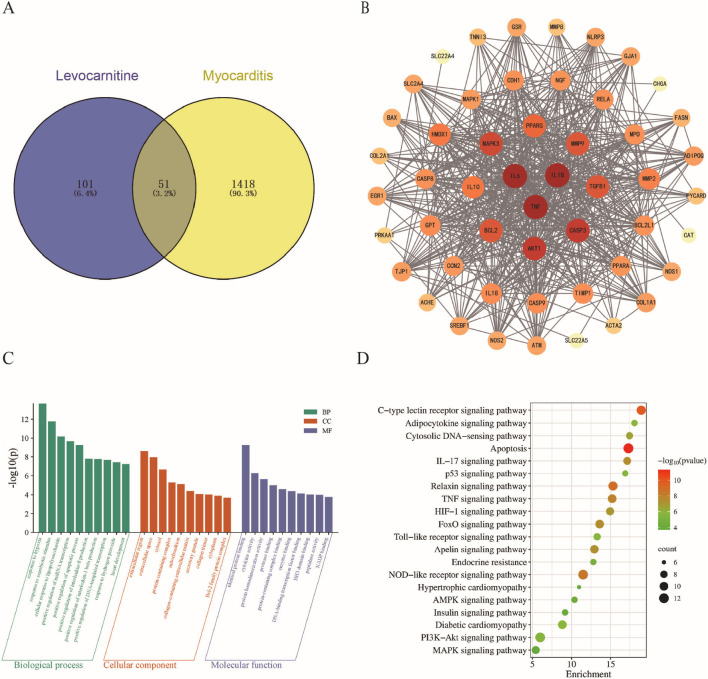
Network pharmacology and enrichment analysis of levocarnitine for myocarditis. **(A)** Venn diagram showing overlapping targets linked to levocarnitine and myocarditis, and **(B)** PPI diagram of potential targets of levocarnitine for myocarditis. **(C)** GO functional enrichment analysis. **(D)** KEGG pathway enrichment analysis. The closer the color is to red, the smaller the P value, indicating more significant enrichment.

To further explore the mechanism of LC in treating myocarditis, we identified LC targets using SwissTargetPrediction, PharmMapper, and CTD databases, resulting in a total of 152 target genes after removing duplicates from the three sources. Next, 1,469 myocarditis-related target genes were filtered and deduplicated from the OMIM, Disgenet, and GeneCards databases. The intersection of LC and myocarditis-related targets was visualized with a Venn diagram, revealing 51 common targets, including TNF, IL1B, IL6, and others (Figure 5A). These 51 targets were imported into the STRING 12.0 database to generate the PPI network, which was then visualized using Cytoscape 3.10.0 software. The PPI network consists of 51 nodes and 579 edges (Figure 5B). The size of the shapes and the intensity of the colors in the graphs are positively correlated with the magnitude of the DC values, where higher DC values indicate that the protein interacts with more other proteins.

The 51 targets of LC acting in myocarditis were analyzed for GO enrichment analysis through the DAVID database. This analysis identified 183 enriched biological processes, including response to hypoxia, positive regulation of apoptosis, IL-1β production, response to hydrogen peroxide, and heart development. Eighteen cellular components, such as mitochondria, extracellular spaces, cytoplasm, and collagen-containing extracellular matrix, are the targets that execute the aforementioned biological processes within these cellular regions. Twenty-one molecular functions reveal the specific biochemical activities of target proteins at the molecular level, including identical protein binding, cytokine activity, protein homodimerization activity, NADP binding, and more (Figure 5C). A total of 108 enriched pathways were obtained from KEGG analysis. Under P < 0.01, pathways not aligned with the research objectives were excluded. Ultimately, 20 pathways were selected for visualization and further analysis, including apoptosis, NOD-like receptor signaling pathway, TNF signaling pathway, and PI3K/Akt signaling pathway, among others (Figure 5D). These systematic network pharmacology analyses indicate that LC likely exerts its therapeutic effects in myocarditis by acting on multiple key targets and signaling pathways.

#### 3.2.2 Results of core target screening and molecular docking

The core targets were identified using the CytoNAC plug-in of Cytoscape software by averaging “DC, CC, BC” as the cutoff value. The results were ranked based on the DC value to highlight IL1B, IL6, TNF, AKT1, CASP3, MAPK3, BCL2, TGFB1, MMP9, and PPARG, which were hypothesized to be key targets of LC treatment for myocarditis (Table 2).

**TABLE 2:** Top 10 hub proteins in the PPI network.

Targets	DC	CC	BC
IL1B	44	0.892857143	106.2754927
IL6	44	0.892857143	106.2754927
TNF	44	0.892857143	106.2754927
AKT1	41	0.847457627	70.99295171
CASP3	41	0.847457627	74.46683719
MAPK3	40	0.833333333	66.18946303
BCL2	39	0.819672131	58.77298498
TGFB1	39	0.806451613	130.4238023
MMP9	39	0.806451613	72.33347805
PPARG	38	0.793650794	88.36940311

DC, degree centrality; CC, closeness centrality; BC, betweenness centrality.

**FIGURE 6:**
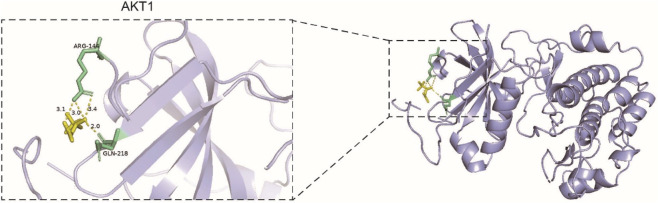
Docking model of LC with AKT1. The yellow region represents the ligand LC, while the purple region corresponds to the AKT1 protein.

The 10 core targets identified from the above screening were molecularly docked with LC. The protein structures of these core targets—IL1B, IL6, TNF, AKT1, CASP3, MAPK3, BCL2, TGFB1, MMP9, and PPARG—were downloaded from the PDB database and docked with LC ligands accordingly. The docking results showed that LC had a good binding affinity with AKT1 (binding energy = −5.4 kcal/mol) (Table 3), which was visualized (Figure 6).

**TABLE 3:** Binding energy of LC to core targets.

Targets	PDB ID	Binding energy (kcal/mol)
IL1B	2nvh	−4.8
IL6	1alu	−4.3
TNF	2e7a	−5.0
AKT1	4ekl	−5.4
CASP3	2xzd	−4.9
MAPK3	3she	−4.6
BCL2	1g5m	−4.6
TGFB1	5vqp	−4.8
MMP9	1itv	−5.2
PPARG	8pbo	−4.9

### 3.3 Effect of LC on Akt and PGC-1α in EAM mice

**FIGURE 7:**
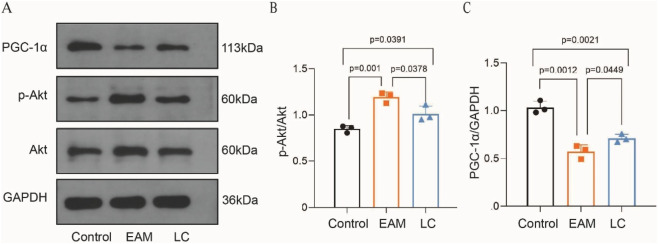
Effect of LC on Akt and PGC-1α in EAM mice. **(A–C)** Changes in Akt, p-Akt, and PGC-1α were analyzed by Western blots, n = 3. Data are expressed as Mean ± SEM with individual data points shown. PGC-1α, peroxisome proliferator-activated receptor gamma coactivator-1α.

To verify the network pharmacology and molecular docking results of LC on myocarditis, we conducted animal experiments (Figure 7A). Compared to the Control group, p-Akt/Akt expression was significantly increased in the EAM mice and decreased after LC treatment (P < 0.05) (Figure 7B). PGC-1α is regarded as a key regulator of mitochondrial metabolism and is essential in controlling cardiac energy metabolism remodeling. The experimental results showed that PGC-1α expression was significantly lower in the EAM mice compared to the Control group, while LC increased PGC-1α expression in myocarditis (P < 0.05) (Figure 7C).

### 3.4 Correlations of p-Akt and PGC-1α with mitochondrial metabolic function

**FIGURE 8:**
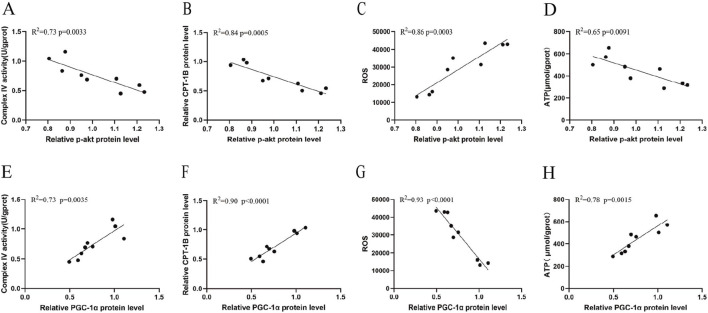
Pearson’s correlation analysis of p-Akt and PGC-1α with mitochondrial metabolic function. Scatter plots showing the correlation between myocardial p-Akt protein expression and **(A)** COX IV activity, **(B)** CPT-1B expression, **(C)** ROS levels, and **(D)** ATP. Scatter plots showing the correlation between myocardial PGC-1α protein expression and **(E)** COX IV activity, **(F)** CPT-1B expression, **(G)** ROS levels, and **(H)** ATP. (n = 9 from 3 groups).

To further investigate the central role of the p-Akt and PGC-1α signaling pathways in regulating cardiac energy metabolism, we performed Pearson correlation analysis between the key signaling molecules and mitochondrial metabolic function indicators. As shown in Figures 8A–D, Akt phosphorylation levels exhibited significant negative correlations with Cox IV activity (R^2^ = 0.73, 95% CI [−0.97, −0.44], P = 0.0033), CPT-1B expression (R^2^ = 0.84, 95% CI [−0.98, −0.64], P = 0.0005), and ATP levels (R^2^ = 0.65, 95% CI [−0.96, −0.30], P = 0.0091), but a positive correlation with ROS levels (R^2^ = 0.86, 95% CI [0.68, 0.98], P = 0.0003). This indicates that the activation of the PI3K/Akt pathway is closely associated with energy deficiency and exacerbated oxidative stress in cardiomyocytes. Furthermore, we analyzed the relationship between PGC-1α and its mitochondrial metabolic functions. As shown in Figures 8E–H, the expression of PGC-1α was positively correlated with Cox IV (R^2^ = 0.73, 95% CI [0.43, 0.97], P = 0.0035), CPT-1B (R^2^ = 0.90, 95% CI [0.77, 0.99], P < 0.0001), and ATP levels (R^2^ = 0.78, 95% CI [0.53, 0.98], P = 0.0015), but negatively correlated with ROS levels (R^2^ = 0.93, 95% CI [−0.99, −0.83], P < 0.0001). This establishes the central role of PGC-1α in coordinating mitochondrial energy production and antioxidant balance.

## 4 Discussion

Myocarditis is a relatively common cause of sudden cardiac death among pediatric and young adults, presenting with heterogeneous clinical manifestations, and it currently lacks specific treatments. As is well known, cardiac diseases are often accompanied by disturbances in cardiac energy metabolism, which can lead to impaired cardiac function and promote disease progression. Mitochondria, frequently called the “energy factories” of cells, are essential in the continuously beating, high-demand cardiomyocytes. The healthy heart has “metabolic flexibility,” enabling mitochondria to adaptively utilize different substrates depending on oxygen levels, substrate concentrations, and enzyme activity to produce ATP that meets cardiac needs, with fatty acids being the most productive substrate (Actis Dato et al., 2024). When myocardial damage causes abnormalities in mitochondrial substrate utilization, oxidative phosphorylation, and a decrease in high-energy phosphate compounds, the myocardium undergoes metabolic remodeling, resulting in cardiac energy deprivation and further exacerbating myocardial damage. Therefore, energy metabolic remodeling plays a key role in the pathological process of myocarditis, and any factors that cause substrate utilization abnormalities or mitochondrial structural dysfunction can affect the onset and progression of myocarditis. Additionally, metabolic disorders lead to pathological lipid accumulation and oxidative stress, causing abnormal cardiac lipotoxicity, which further worsens cardiac metabolic damage (Fonseka et al., 2025). LC, a substance that facilitates fatty acid transport and regulates mitochondrial energy metabolism, has been employed clinically in myocarditis management (Xu et al., 2018), though its exact therapeutic mechanism remains unclear. This study aims to investigate the therapeutic effects of LC on energy metabolism remodeling in myocarditis by applying LC to an EAM mouse model and exploring further potential signaling pathways through network pharmacology and molecular docking.

The study demonstrated that LC improved cardiac insufficiency and reduced myocardial inflammatory infiltration in EAM mice. Further research was conducted to explore the effects of LC on energy metabolism remodeling in myocarditis. The experimental results revealed for the first time that LC alleviated mitochondrial structural damage in the myocardium of EAM mice and regulated mitochondrial substrate utilization and respiratory chain function. Mitochondrial fatty acid β-oxidation is the primary metabolic pathway for myocardial energy production. Long-chain fatty acids cross the myocardial cell membrane via transporters (such as H-FABP), bind with CoA, and are then converted into acylcarnitine by CPT-1 on the outer mitochondrial membrane to enter the mitochondria for β-oxidation (Lopaschuk et al., 2021). H-FABP has been widely used for early diagnosis and prognosis assessment of cardiovascular diseases. When myocardial ischemia-hypoxia damage occurs, H-FABP is rapidly released into the bloodstream, making it a common early biomarker for myocardial injury in clinical practice (Ye et al., 2018; Moon et al., 2021). The experiment also confirmed that LC decreased serum H-FABP release in EAM mice, indicating diminished myocardial damage. Moreover, since H-FABP is a protein involved in fatty acid transport, its reduced release indirectly suggests an improvement in cardiomyocyte fatty acid utilization. CPT-1B, the major CPT-1 isoform expressed in the myocardium, is the rate-limiting enzyme for cardiac fatty acid β-oxidation (Bonnefont et al., 2004). OCTN-2 represents a crucial transporter involved in transporting carnitine across the cellular membrane. Grube et al. observed a significant reduction in OCTN2 expression, along with elevated plasma carnitine levels, diminished fatty acid β-oxidation, and a correlation between OCTN2 expression and cardiac functional parameters (such as LVEF) in the CVB_3_-induced cardiomyopathy model (Grube et al., 2011). Our results demonstrated that CPT-1B and OCTN-2 expression were upregulated in EAM mice after LC treatment, suggesting that LC promotes energy production by improving fatty acid transport. Additionally, the accumulation of FFA and LAC decreased in EAM mice after LC intervention, further confirming that LC boosts fatty acid metabolism, inhibits the compensatory upregulation of anaerobic glycolysis, indicating a shift in myocardial substrate utilization from glycolysis back toward fatty acid oxidation. Furthermore, we observed that LC increased the activity of COX IV, a key enzyme in the mitochondrial respiratory chain, and elevated ATP generation while attenuating ROS accumulation. These findings indicate that LC can alleviate oxidative phosphorylation and oxidative stress disorders in myocarditis, improving respiratory chain efficiency, thereby improving mitochondrial bioenergetic function in EAM mice. In summary, we believe that LC enhances energy metabolism remodeling in myocarditis by promoting fatty acid transport and metabolism, alleviating mitochondrial structural damage, increasing respiratory chain enzyme activity, inhibiting oxidative stress, and boosting ATP production, ultimately helping to alleviate heart failure in EAM mice.

There have been no studies on the mechanism of LC in treating myocarditis. To explore this potential mechanism more thoroughly, we conducted GO and KEGG pathway enrichment analyses on 51 protein target genes of LC acting on myocarditis obtained through network pharmacology. The GO functional analysis indicated that LC might exert therapeutic effects by participating in biological processes such as hypoxia, IL-1β production, and responses to hydrogen peroxide. Early myocardial ischemia and hypoxia trigger compensatory mechanisms, but prolonged hypoxic damage increases pro-inflammatory cytokines (IL-1β, IL-6, and TNF-α) and reactive species (O2·−, ·OH, and H2O2), and shifts metabolism from fatty acid oxidation to glycolysis, further worsening cardiac hypertrophy, myocarditis, and heart failure (Adzika et al., 2023; Ndzie Noah et al., 2024). Existing research shows that LC can reduce myocardial injury by alleviating inflammatory responses and oxidative stress (Emran et al., 2021), which aligns with our findings. Cardiac inflammatory response and mitochondrial dysfunction mutually drive each other, acting as a key pathological link in the progression of cardiac diseases (Zuo et al., 2024). Hahn et al. (Hahn et al., 2014) found that pro-inflammatory factors (such as IL-1β, IL-6, and TNF-α) influence mitochondrial biogenesis by regulating the NAD^+^/NADH ratio, oxidative stress, and kinetics. The results of CC annotation in this study also suggest that LC may regulate the processes mentioned above by acting on cellular components such as mitochondria. Therefore, GO analysis further confirms that LC can alleviate the pathological process of myocarditis by regulating mitochondrial function in cardiomyocytes.

Further KEGG analysis revealed that the 51 acquired protein target genes were associated with the PI3K/Akt signaling pathway. Topological analysis identified the top 10 key targets: IL1B, IL6, TNF, AKT1, CASP3, MAPK3, BCL2, TGFB1, MMP9, and PPARG. To validate the network pharmacology predictions and better understand protein-ligand interactions, the molecular docking technique was applied to predict the direct binding patterns and binding free energies between the compound and target proteins, and to evaluate the affinity between LC and key target proteins. The results demonstrated that LC exhibits good binding affinity with Akt. The PI3K/Akt signaling pathway regulates various biological functions by activating downstream effectors that influence cell cycle transitions and proliferation (Ghafouri-Fard et al., 2022). Extensive evidence demonstrates that PI3K/Akt is a central regulator of cardiomyocyte function and survival, involving multiple aspects of inflammatory regulation, apoptosis, oxidative stress, and cardiac hypertrophy, and it plays an important regulatory role in the progression of cardiovascular disease (Cheng et al., 2021; Qin et al., 2021; Ren et al., 2020). It has been demonstrated that PI3K/Akt signaling is significantly altered in the autoimmune myocarditis model, and inhibiting this pathway reduces myocardial inflammatory responses and protects cardiac function (Shi et al., 2017; Zhang et al., 2019). Our animal experiment also confirmed increased p-Akt/Akt expression in myocarditis, while LC inhibited cardiac Akt phosphorylation. Based on the convergent evidence from enrichment analysis, molecular docking, and animal validation, we have identified Akt as the key target of LC against myocarditis: it exhibits optimal topological centrality in the PPI network, demonstrates the strongest binding affinity with LC, and serves as a signaling hub in the PI3K/Akt pathway that is closely associated with the pathological progression of myocarditis. Therefore, we speculate that LC may regulate cardiomyocyte mitochondrial function by inhibiting the PI3K/Akt signaling, thereby influencing the energy metabolism remodeling in myocarditis.

The Akt signaling pathway plays a critical regulatory role over PGC-1α in the cardiovascular system. In vascular smooth muscle cells, angiotensin II activates Akt to mediate PGC-1α phosphorylation at Ser570. This modification facilitates its binding to histone acetyltransferases and ultimately inhibits PGC-1α′s transcriptional activity (Xiong et al., 2010). Similarly, transgenic mice with cardiac-specific expression of constitutively active Akt exhibited a significant downregulation of PGC-1 mRNA levels (Cook et al., 2002). PGC-1α, a key molecule that controls the number and function of mitochondria to regulate energy demands, is significantly upregulated in the heart at birth. It mediates cardiac development, regulation of oxidative phosphorylation, fatty acid metabolism, and reactive oxygen species homeostasis (Rowe et al., 2010; Rog-Zielinska et al., 2015; Lai et al., 2008). Several studies have demonstrated that PGC-1α can activate CPT-1B expression to regulate myocardial fatty acid β-oxidation and mitochondrial respiration function (Sharma et al., 2009; Papatheodorou et al., 2022). It was found that the expression levels of various genes involved in PGC-1α, fatty acid β-oxidation, and oxidative phosphorylation signaling pathways decreased in viral myocarditis, and these levels were significantly negatively correlated with the expression of pro-inflammatory cytokines (TNF-α) (Remels et al., 2018). Therefore, dysregulation of PGC-1α can severely disrupt cardiac metabolic homeostasis and cause various cardiovascular diseases (Qian et al., 2024). Wende et al. proposed that activation of Akt may limit cardiac “metabolic flexibility” by inhibiting the expression of PGC-1α, shifting substrate preference toward glucose utilization while impairing mitochondrial oxidative capacity and ATP production (Wende et al., 2015). We also observed reduced Akt phosphorylation and increased PGC-1α expression in EAM mice myocardium after LC treatment. We speculate that LC may improve energy metabolism remodeling in EAM myocardium by regulating the PI3K/Akt pathway, and this may be associated with increased PGC-1α expression. Correlation analysis confirmed a significant negative association between p-Akt and PGC-1α levels (R^2^ = 0.68, 95% CI [−0.96, −0.36], P = 0.0061; Supplementary Figure S1). Further analyses demonstrated that PGC-1α expression was positively correlated with ATP levels, COX IV activity, and CPT-1B expression, and negatively correlated with oxidative stress. Collectively, our findings provide new experimental and statistical support for a novel mechanistic framework in which LC ameliorates energy metabolism remodeling by suppressing PI3K/Akt signaling to upregulate PGC-1α.

This study provides innovative research perspectives and a clinical rationale for LC in modulating energy metabolism remodeling in myocarditis. As an endogenous compound, LC has favorable pharmacokinetics and targets the heart via active uptake against plasma gradients mediated by the OCTN2 transporter (Wang et al., 2018). The optimal dose identified in our murine model (200 mg/kg∙d, Supplementary Figure S2) corresponds to common clinical doses (adults: 1–3 g/d; children: 50–100 mg/kg∙d), suggesting potential translational relevance of its metabolic benefits. While its common side effects (such as gastrointestinal discomfort and fishy odor) are reversible and dose-dependent, chronic administration poses a specific risk because LC is metabolized into trimethylamine-N-oxidase (TMAO)—a pro-atherogenic metabolite that paradoxically counteracts its cardioprotective role (Elantary and Othman, 2024). The demonstrated efficacy of LC in ameliorating myocardial energy metabolism remodeling in animal models, together with its established clinical profile, justifies conducting a Phase I clinical trial to evaluate its metabolic therapeutic potential in myocarditis patients, while maintaining TMAO monitoring during extended administration.

This study has certain limitations. Although the EAM mouse model is frequently used in myocarditis research because of its similarities with the pathological features and immune responses observed in human myocarditis (Ml et al., 2021), its pathophysiological process mainly involves autoimmune-mediated myocardial injury, which differs from the viral myocarditis commonly encountered in clinical practice. Additionally, future detailed validation of the PI3k/Akt and PGC-1α in myocarditis treatment by LC was not conducted in animal models to clarify their causal relationships in therapy, and the effects of other signaling pathways could not be entirely ruled out yet. Future research should focus on interventions targeting the PI3K/Akt pathway and/or constructing PGC-1α gene-edited mice to verify the mechanisms by which LC improves energy metabolism remodeling in myocarditis.

## 5 Conclusion

The study combines animal experiments, network pharmacology, and molecular docking to reveal, for the first time, the beneficial effects of LC on energy metabolism remodeling in myocarditis, while preliminarily identifying the underlying molecular mechanisms. The results showed that LC can improve fatty acid metabolism, alleviate mitochondrial structural damage and respiratory chain dysfunction, inhibit oxidative stress, promote ATP production, and alleviate cardiac insufficiency in myocarditis mice. We speculate that LC may improve myocardial metabolic remodeling by inhibiting PI3K/Akt signal transduction, which could be linked to increased expression of the mitochondrial key regulator PGC-1α. This study provides innovative methods and insights into therapeutic targets for LC-mediated regulation of energy metabolism remodeling in myocarditis.

## Data Availability

The original contributions presented in the study are included in the article/Supplementary Material; further inquiries can be directed to the corresponding author.
