# Supplementary figures and images for: Levocarnitine improves cardiac energy metabolic remodeling in myocarditis mice
Source: Front Pharmacol. 2026 Jan 9;16:1706936. doi: 10.3389/fphar.2025.1706936 (PMC12827101; doi:10.3389/fphar.2025.1706936)

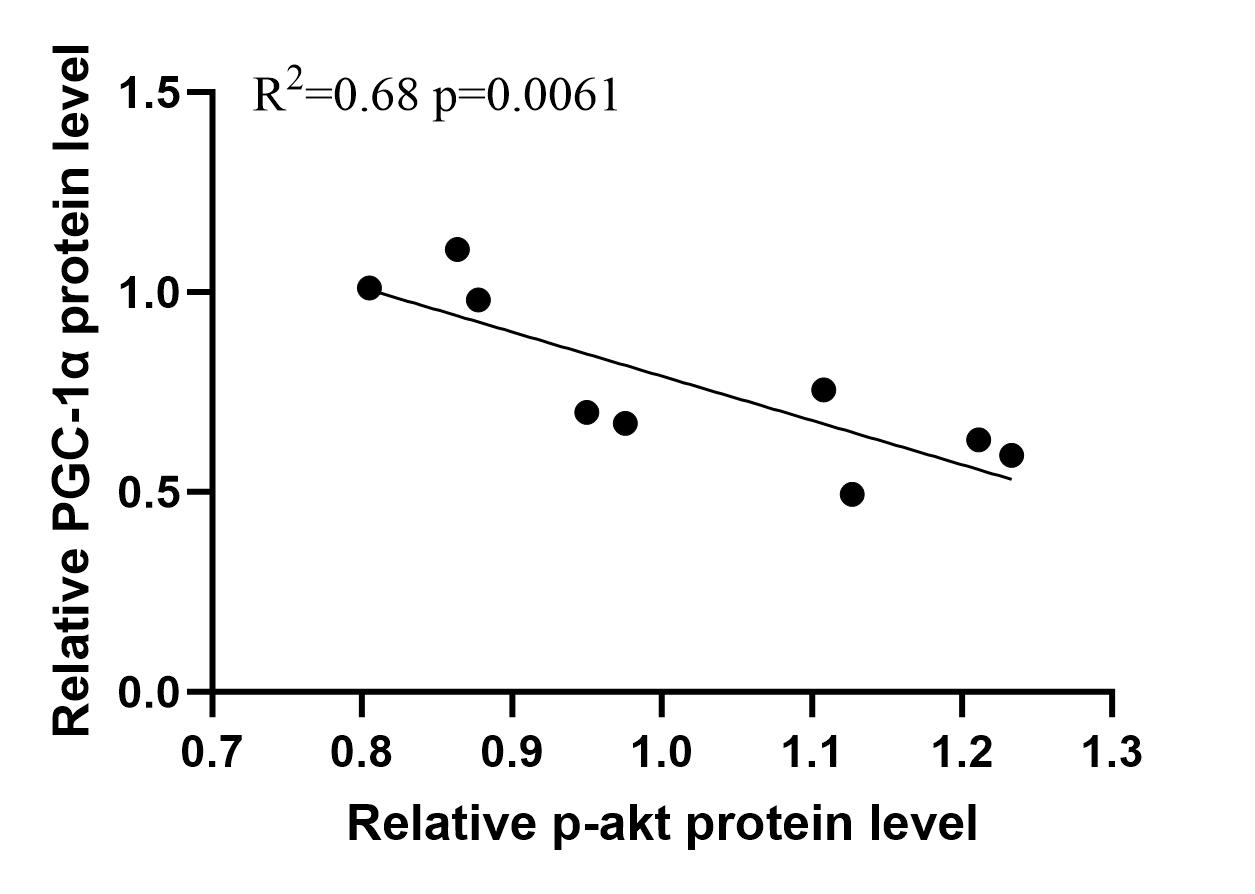

Supplement: Supplementary file 1 [file Image1.JPEG]

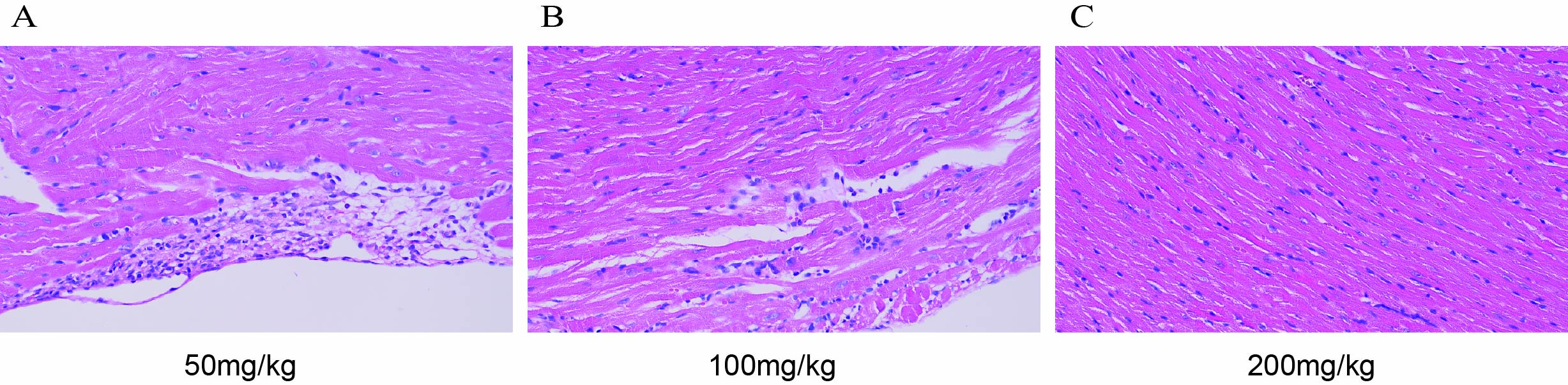

Supplement: Supplementary file 2 [file Image2.JPEG]
